# Nanocarbon-Based Mixed Matrix Pebax-1657 Flat Sheet Membranes for CO_2_/CH_4_ Separation

**DOI:** 10.3390/membranes13050470

**Published:** 2023-04-28

**Authors:** Athanasios N. Vasileiou, George V. Theodorakopoulos, Dionysios S. Karousos, Mirtat Bouroushian, Andreas A. Sapalidis, Evangelos P. Favvas

**Affiliations:** 1 Institute of Nanoscience and Nanotechnology, National Center for Scientific Research “Demokritos”, Aghia Paraskevi, 15341 Attica, Greece; purinikos@hotmail.com (A.N.V.); d.karousos@inn.demokritos.gr (D.S.K.); a.sapalidis@inn.demokritos.gr (A.A.S.); 2 School of Chemical Engineering, National Technical University of Athens, 9 Iroon Polytechniou Street, Zografou, 15780 Athens, Greece; mirtatb@central.ntua.gr

**Keywords:** mixed matrix membranes (MMMs), supported thin films, carbon nanofillers, Pebax-1657, CNTs dispersion, GNPs dispersion, gas separation

## Abstract

In the present work, Pebax-1657, a commercial multiblock copolymer (poly(ether-block-amide)), consisting of 40% rigid amide (PA6) groups and 60% flexible ether (PEO) linkages, was selected as the base polymer for preparing dense flat sheet mixed matrix membranes (MMMs) using the solution casting method. Carbon nanofillers, specifically, raw and treated (plasma and oxidized) multi-walled carbon nanotubes (MWCNTs) and graphene nanoplatelets (GNPs) were incorporated into the polymeric matrix in order to improve the gas-separation performance and polymer’s structural properties. The developed membranes were characterized by means of SEM and FTIR, and their mechanical properties were also evaluated. Well-established models were employed in order to compare the experimental data with theoretical calculations concerning the tensile properties of MMMs. Most remarkably, the tensile strength of the mixed matrix membrane with oxidized GNPs was enhanced by 55.3% compared to the pure polymeric membrane, and its tensile modulus increased 3.2 times compared to the neat one. In addition, the effect of nanofiller type, structure and amount to real binary CO_2_/CH_4_ (10/90 vol.%) mixture separation performance was evaluated under elevated pressure conditions. A maximum CO_2_/CH_4_ separation factor of 21.9 was reached with CO_2_ permeability of 384 Barrer. Overall, MMMs exhibited enhanced gas permeabilities (up to fivefold values) without sacrificing gas selectivity compared to the corresponding pure polymeric membrane.

## 1. Introduction

In recent years, gas separation, such as N_2_ production, H_2_ recovery, CO_2_ capture and natural gas sweetening, is achieved with polymeric membranes, an efficient process competing sufficiently with well-established separation processes such as adsorption, extraction and cryogenic distillation [1]. Membranes offer easy fabrication and scalability, low capital and operating cost, operation simplicity, low energy consumption and maintenance, mechanical reliability and small carbon footprint. All these features render them a promising method in high-performance gas-separation applications [2]. However, the gas-separation performance of polymeric membranes is frequently restricted by the Robeson’s trade-off upper bound [3]. Polymers with high permeability exhibit low selectivity and vice versa [3,4].

To address this, nanotechnology is used in membrane science, creating a new type of nanoengineered materials, where the membrane properties are modified in the direction of improving the filtration/separation processes [5]. Among numerous different modification processes, such as polymer blending [6], cross-linking [7], surface modification [8], surfactant/salt addition [9,10], high-energy x-ray irradiation [11], thermal treatment, annealing post-treatment [12] and immobilization of specific ligands [13], the preparation of mixed matrix membranes, by adding functional nanomaterials into polymer matrix, is a promising technique for the preparation of membranes with improved separation properties [14].

Hence, nanoporous carbon-based nanofillers are incorporated into polymer matrices as a dispersed phase forming mixed matrix membranes (MMMs), which subsequently can provide an increase in selectivity and permeability simultaneously [15]. This fact makes them promising types of filler material in high-performance gas-separation applications [2,15,16] by combining the gas-separation properties of inorganic, e.g., molecular sieve, particles with the advantageous properties of a polymeric matrix [17]. However, MMMs still present shortcomings such as physical aging, plasticization and also low processing capacity [18]. Criteria for the selection of membrane materials, both for polymer matrices and added fillers, are key and significant parameters of preparing highly selective, highly permeable and overall efficient membranes [19]. A crucial factor for this selection is the requested separation, which we are interested in working on. For example, in the case of CO_2_/CH_4_ separation, a criterion for polymeric material selection is the existence of a high affinity to polar molecules, such as CO_2_, compared to nonpolar molecules, such as CH_4_. The requirements for good mechanical behavior and of course high resistance to thermal and chemical strains also remain. Among many other polymeric membrane materials, such as polysulfones, polyimides, polyamides, polyesters and cellulose acetate, poly(ether-block-amide) (Pebax) is classified as one material which satisfies adequately the abovementioned requirements [20,21]. Based on the fact that one of the approaches for overcoming the undesirable trade-off relationship between permeability and selectivity is to use polymeric materials, which combine the flexibility of polymers such as polyethers (PEs) and the stability of rigid polymers such as polyamides (PAs), polyether block amide copolymers (PEBAs) are classified as some of the most promising materials for CO_2_ selective membrane fabrication [22]. The PE segment in the polymer structure is responsible for permeability of penetrants through the membrane owing to high mobility of chains in the polymer matrix, whereas the PA segment is impermeable [21].

The prospects offered by this material’s family have attracted the interest of many research groups worldwide, and during the last two decades many encouraging studies have been conducted based on Pebax gas-selective membranes in literature. Some relevant works and their main evidence in chronological order are reported in the following. 

Liu et al. [23], in 2014, prepared and studied a series of hydrogel membranes from chitosan (CS)/polyether-block-amide (Pebax) blends. These membranes exhibited high CO_2_ permeabilities along with good operation stabilities. Specifically, the membrane with a mass ratio of CS to Pebax equal to 1:1 showed a very high CO_2_ permeability of about 2880 Barrer and a CO_2_/CH_4_ separation factor of ~23 at 85 °C. The gas permeability/selectivity experiments were performed in the presence of relative humidity >98%, which facilitated transport membrane separation. Estahbanati et al. [24], in 2017, incorporated features of the Pebax-1657 copolymer by adding [BMIM][BF4] ionic liquid to increase the permselectivity of the membranes for CO_2_/light gas separation, because the CO_2_ solubility in ionic liquids (ILs) increases with pressure increment and temperature decrement. In addition, due to the high affinity of CO_2_ in both polymer and IL, both CO_2_ permeability and selectivity increased simultaneously with increasing IL content. Their hypothesis was confirmed by gas permeation results, where at 35 °C and 10 bar, the CO_2_ permeability increased from 110 Barrer for neat Pebax to 190 Barrer in the blended membrane containing 50 wt.% IL. The corresponding CO_2_/CH_4_ and CO_2_/N_2_ selectivities increased from 20.8 to 24.4 (about 17%) and from 78.6 to 105.6 (about 34%), respectively. 

Zhang et al. [25], in 2018, reported that by microphase separation the polymer chain packing density changes along with the molecular separation efficiency within the composite halloysite nanotube (HNT)/Pebax-1657 membranes. This fact was taken into consideration in the case of a thin composite Pebax membrane, through the controllable self-assembly of one-dimensional halloysite nanotubes (HNTs, up to 0.2 wt.% towards the casting solution) within the thin film via a solution casting technique. This change in crystallization of the polyamide component, which was induced at the HNT surface, provided the composite membrane an ultrahigh CO_2_/N_2_ selectivity of up to 290 combined with a moderate CO_2_ permeability of 80.4 Barrer. In another work, in 2019, Farashi et al. [21] studied Pebax-1657 mixed matrix membranes by using different contents of aluminum oxide (Al_2_O_3_) (0, 2, 4, 6 and 8 wt.%) in the polymer matrix (Pebax). Permeances of pure CO_2_ and CH_4_ gases were measured in the range of pressures between 3 and 15 bar at 25 °C. The results revealed better separation efficiency (both CO_2_ permeability and CO_2_/CH_4_ selectivity) of the nanocomposite membranes than the pristine membrane. For example, the CO_2_ permeability and ideal CO_2_/CH_4_ selectivity values for the neat membrane at the pressure of 3 bar were 123.46 Barrer and 21.21, respectively, whereas those values for the membrane comprising 8 wt.% of Al_2_O_3_ were 159.27 Barrer and 24.73, respectively.

In addition to the polymer matrix selection, the selection of the specific kind of filler membrane materials is another route for improving the selectivity performance of the membrane. Carbon-based nanomaterials are reported as promising filler materials for producing improved mixed matrix membranes for gas-separation applications [5]. Among others, multi-walled carbon nanotubes (MWCNTs) and graphene nanoplatelets (GNPs) [26,27] are two types of nanocarbons, nanoscale carbon derivative materials, which can provide the requested improvement of both permeability and selectivity performance of the derivative mixed matrix membrane if they are used as membrane filler materials. The fact that both MWCNTs and GNPs are relatively cheap and easily available materials combined with their good physicochemical properties have rendered them particularly popular in composite membrane technology. Among others, some of the advantages of these two nanomaterials are (1) the facile production methods, (2) the ability for surface modification using wet chemistry, (3) their dispersibility using sonication, (4) their conductive properties and (5) their large specific surface areas [28].

In current work, Pebax-MH1657, a commercial multiblock copolymer (poly(ether-block-amide)) with remarkable CO_2_ separation properties was selected as the base polymer of MMMs. In addition, both raw and treated MWCNTs and GNPs were used as membrane filler materials for producing composite membranes of 0.7, 3.0 and 5.3 wt.% in carbon nanofillers relative to the polymer content. The characterization and the performance-evaluation of thirteen produced membranes were carried out by means of SEM, FTIR, gas mixture permeability evaluation for CO_2_/CH_4_ (10/90), contact angle measurements and mechanical tests.

## 2. Materials and Methods

### 2.1. Materials

Pebax MH 1657 (containing approximately 60 wt.% polyether segments and 40 wt.% polyamide segments) was purchased from Arkema S.A., France. Ethanol was purchased from VWR International Ltd., Lutterworth, UK. All of the above chemicals were of analytical grade and were used without further purification. Ultrapure water (Milli-Q, 18 MΩ·cm) was used throughout this study. The carbon nanofillers were provided from our colleagues from FutureCarbon GmbH, Bayreuth, Germany.

For the preparation of flat sheet Pebax-1657-based mixed matrix membranes, a dispersion of raw and treated (plasma or oxidized) multi-walled carbon nanotubes (MWCNTs) and graphene nanoplatelets (GNPs) was incorporated into the polymeric matrix in order to improve the gas-separation performance and the polymer’s structural properties.

The raw GNPs were produced by a water-based milling process and for the wet chemical treatment of GNPs, KMnO_4_ was employed as oxidizing agent. In addition, the raw MWCNTs were produced by chemical vapor deposition of hydrocarbon gas on iron-based catalysts, and for the functionalization of MWCNTs, plasma treatment was implemented (optimum operational parameters: He with 500 W plasma power for 10 min exposure time and O_2_ with 500 W for 70 min exposure time).

**Figure 1:**
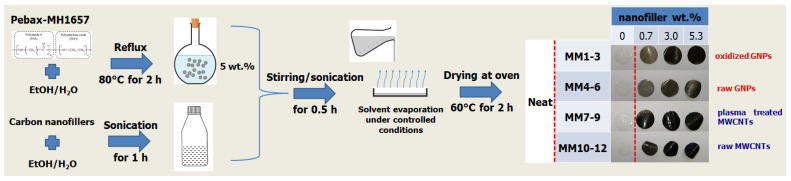
Schematic representation of all intermediate steps for Pebax-1657-based MMM production.

Dispersions of all twelve different MMM systems were formed by the solution blending method with an additional dispersion step to avoid agglomeration. The obtained homogeneous solutions were poured into Petri dishes, and solvent evaporation was performed under controlled atmospheric conditions. Finally, the films were dried in an oven at 60 °C for 2 h in order to remove any residual solvent. The overall membrane preparation process is illustrated in Figure 1.

The status of dispersion quality was evaluated directly by optical determination of the solutions and the prepared membranes, as well as indirectly mainly by mechanical strength testing and microscopic analysis. After preparation, the membranes were dried, and permeation properties were analyzed for CO_2_ and CH_4_.

### 2.2. Instrumentation-Characterization 

The composite carbon-based Pebax-1657 membranes were evaluated concerning their CO_2_/CH_4_ selectivity and permeability performance in a flow selectivity apparatus in conjunction with high-sensitivity gas chromatography. In addition, the prepared membranes were characterized by Fourier transform infrared spectroscopy (FTIR) conducted on a Nicolet Magna-IR Spectrometer 550 (Thermo Fisher Scientific, Waltham, MA, USA). The morphological characterization of selected samples was investigated by scanning electron microscopy (SEM) in a JEOL JSM-7401F instrument (Tokyo, Japan). The membranes’ mechanical properties (Young’s modulus, ultimate tensile strength and tensile elongation) were determined in a Thümler GmbH Tensile Tester Model (Roth, Germany) equipped with a PA6110 Nordic Transducer load cell with a maximum force of 250 N [29]. The specimens were prepared according to ASTM D882. Dynamic contact angle (CA) measurements of water/membrane interfaces took place using the Krüss DSA30S optical contact angle measuring instrument (Krüss GmbH, Hamburg, Germany).

### 2.3. Membrane Preparation

The first step of the membrane preparation process was the blending of the two solutions, “A” and “B”. The “A” solution was prepared after solving the Pebax-MH1657 pellets, 5 wt.%, into EtOH/H_2_O (70/30 wt.%) solvent, whereas the “B” solution was derived by dispersing the carbon nanofiller into the same solvent (EtOH/H_2_O, 70/30 wt.%). The solution “A” was first refluxed at 80 °C for 2 h, and solution “B” was sonicated for 1 h. The final solution obtained after the mixing of both prepared solutions was stirred and sonicated for half an hour before it was poured into a flat glass Petri dish. Subsequently, the solvent was evaporated overnight at room temperature, and finally the films were dried in an electrical oven at 60 °C for a period of time of about two hours. In Table 1, all the studied cases for the MMMs preparation are presented.

### 2.4. Gas Permeability/Separation Measurements under Continuous Flow Conditions

**Figure 2:**
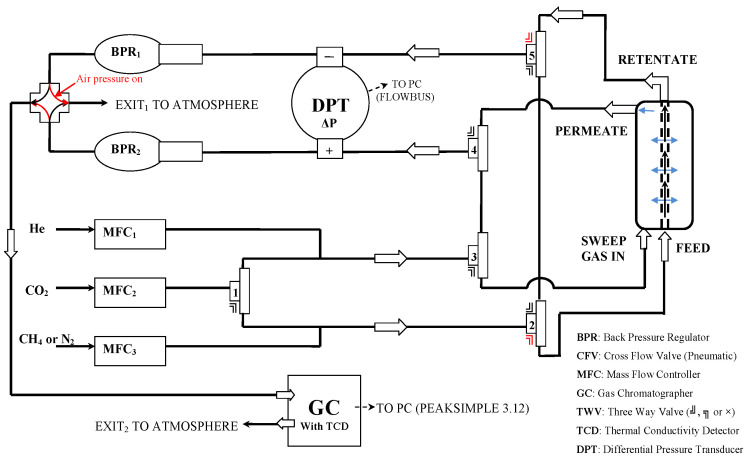
Scheme of laboratory apparatus for gas-separation experiments under continuous flow [32].

Gas permeability and selectivity evaluation was performed by the “flow method” using the gas chromatography (GC) technique. The gas permeance values were measured in the apparatus presented in Figure 2, where the permeate stream was directed to a highly sensitive gas chromatography instrument, and the permeance coefficient was calculated by the integration of the recorded GC peak [30]. As a carrier gas, helium was used (Figure 2). The experimental setup for the permeance measurements, using gas chromatography analysis, has been described in detail previously [31]. Using this setup, mixture selectivity experiments of 10/90 (mole concentration) for CO_2_/CH_4_ were performed.

All thirteen flat sheet membranes, each one with an effective permeation area of about 5.3 cm^2^, were successively inserted into a metallic (bronze) membrane housing. Each membrane was placed into the cell and thoroughly degassed for at least 24 h at 10^−6^ mbar and 80 °C before permeance/selectivity measurements.

The 10/90 CO_2_/CH_4_ (mole concentration) gas mixture was introduced to the feed side of the membrane, whereas helium was used as the sweep gas on permeate side. Mass flow controllers (Brooks Instruments 0–50 mL/min) were used to define the flow rates of each gas. In the retentate side, the pressure was controlled by a backpressure regulator, while the permeate side was maintained at atmospheric pressure. Transmembrane pressure was recorded using a differential manometer. An 8610C gas chromatograph equipped with high-sensitivity TCD and FID detectors was used for analysis of both gas lines.

The selectivity coefficients were calculated according to the following equation [33]:

(1) $$S=\frac{A_{\left(gas1/perm\right)}/A_{\left(gas2/perm\right)}}{A_{\left(gas1/feed\right)}/A_{\left(gas2/feed\right)}}$$

where $A_{\left(gas1/perm\right)}$, $A_{\left(gas1/feed\right)}$ and $A_{\left(gas2/perm\right)}$, $A_{\left(gas2/feed\right)}$ are the peak surfaces for the permeate and feed gas streams, respectively.

## 3. Results and Discussion

### 3.1. Morphology of Nanofillers/Prepared MMMs

**Figure 3:**
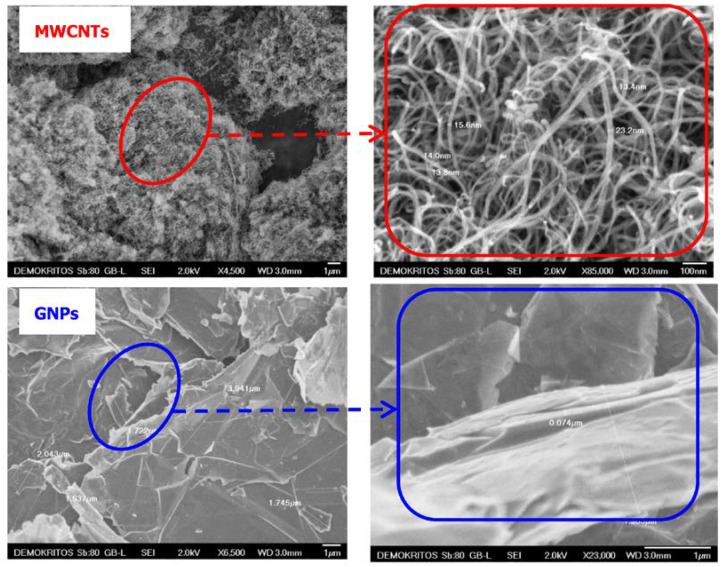
SEM images of raw multi-walled carbon nanotubes (MWCNTs) and graphene nanoplatelets (GNPs).

High-purity carbon nanotubes were produced by catalyst-assisted chemical vapor deposition [34] and were treated as described in Section 2.1. The GNPs were produced following the water-based milling process [35] and were oxidized using KMnO_4_ as oxidizing agent (see Section 2.1). In the following Figure 3, SEM micrographs illustrate the morphology of MWCNTs and their interwoven and entangled arrangement. They appeared in the form of ribbon complexes with no sign of any impurities, and their outer diameter ranged between 13 and 23 nm. The GNPs fillers have a wide range of dimensions both in thickness and lateral dimensions, which fluctuate from 2 to 5 μm and from 50 to 100 nm, respectively. In addition, GNPs have a high purity and well-defined structure of uniform flakes.

**Figure 4:**
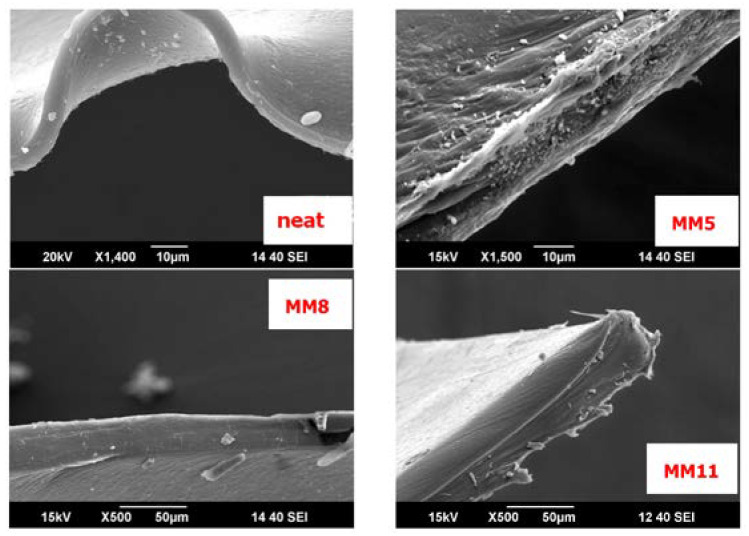
Morphology of dense neat Pebax-1657 and nanocarbon-based Pebax-1657 mixed matrix membranes.

Figure 4 summarizes the SEM micrographs of four selected membranes. The selected membranes are the neat Pebax-MH1657 membrane, the MM5 sample (3 wt.% of raw GNPs relative to polymer content), the MM8 sample (3 wt.% of plasma-treated MWCNTs) and the MM11 sample (3 wt.% of raw MWCNTs). SEM images illustrate two main characteristics of the prepared membranes, namely that they show dense structure without any pinholes and that their thickness ranges from about 8 to 90 μm. Both carbon nanotubes and GNPs are not visible in the cross-sectional images of the three selected mixed matrix membranes in the presented range of magnitude, in which no significant differences in the matrix are observed.

This may be sufficient proof for the homogeneous dispersion of the filler in the polymer matrix, without observable agglomerates or obvious defects in the matrix, indicating the good affinity and adhesion between the polymer and nanofillers. The existence of some white “dots”, especially on the MM5 membrane, can be attributed to dust, which sticks to the surface after membrane cutting preparation with liquid nitrogen as a cooling agent. The membranes are extremely flexible, which becomes obvious in the SEM image of the neat sample (curved membrane). 

### 3.2. FTIR Analysis

**Figure 5:**
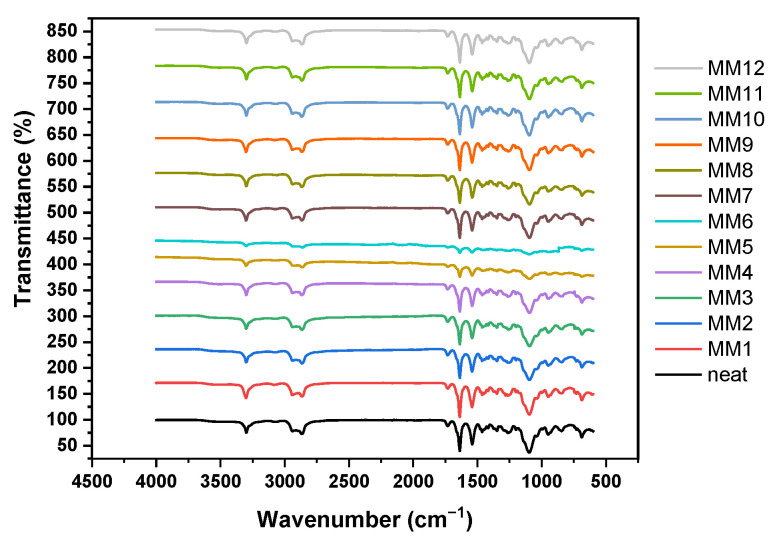
FTIR spectra of the thirteen Pebax-1657 membranes, the neat and the twelve mixed matrix membranes.

The FTIR spectra of the thirteen Pebax-1657-based membranes are shown in Figure 5. All samples exhibit very similar spectra. The peak at 3294 cm^−1^ indicates the presence of N-H amide group [36] and the peaks at 2938 and 2864 cm^−1^ the existence of the aliphatic –C–H groups and the vibrations of the δ(C–H) and ν(C–H) [37].

The characteristic peaks at 1640 and 1544 cm^−1^ correspond to the hydrogen-bonded amide peak and to the C–O stretching band, respectively. The two characteristic peaks at 1731 and 1099 cm^−1^ correspond to C=O (carbonyl group) and C–O-C (ether group) stretching vibrations in the pure Pebax-1657 structure [38].

### 3.3. Water Contact Angle Measurements

The water contact angle (WCA) measurements were performed employing a Krüss DSA30S instrument as mentioned. The CA measuring instrument has a range of 180° for surface tensions ranging from 0.01 to 2000 mN/m. A digital image followed by the calculated droplet’s contact angle is recorded automatically by the Advance-Krüss software. The instrument provides remarkable reproducibility and high accuracy of measurement [39]. During the measurement, at any equilibrium stage of the drop/surface system, a calculated contact angle is recorded automatically.

The affinity of the membranes’ surfaces to water was assessed by the equilibrium contact angle of all studied samples through the contact angle measurements, as shown in Table 2.

The presented value of each sample is the average value of five measurements from different spots of the membranes’ surfaces. It is obvious that in all cases of mixed matrix membranes, surface hydrophilicity was lower compared to the neat Pebax-1657 membrane. Specifically, the WCA is 63.4° for the neat Pebax-1657 membrane, while for the derived MMMs it fluctuated between 69 and ~108°. The same behavior was also previously observed for cross-linked Pebax membranes, where higher grade of crosslinking resulted in an analogous increase in surface hydrophobicity [40]. Similarly, this trend has also been presented in a study regarding MWCNTs/Pebax MMMs [41].

**Figure 6:**
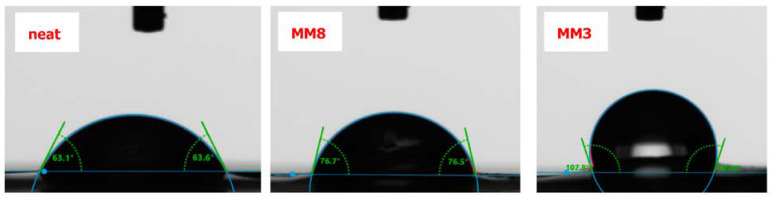
Characteristic images of water contact angles on three membrane surfaces.

In all four groups of mixed matrix membranes (relative to filler’s type) a common feature was noticed: An increase in filler concentration leads to a surface hydrophilicity reduction. In particular, the GNP fillers affect more intensely the membranes’ hydrophilicity, as these nanomaterials render the property of hydrophobicity sturdier than the MWCNTs. This can be attributed to shape and dimensions (see Section 3.1) of the robust GNPs. The edges of GNP flakes protruding from the surface form a kind of comb at the membrane’s surface, which increases its roughness. The existence of the GNPs’ edges/wrinkles is probably more in line with a Cassie–Baxter wetting state than a Wenzel state [42], and therefore the surface hydrophilicity decreases. The higher roughness leads to reduction in water wettability. Furthermore, as observed in Table 2, the measured higher WCA values correspond to the modified nanofillers (both cases of MWCNTs and GNPs) and not the raw nanofillers, providing good evidence for their better dispersion and compatibility with the polymeric matrices. The difference of the surface hydrophilicity is apparent in the three selected water contact angle images, which are presented in Figure 6.

### 3.4. Mechanical Properties

The mechanical behavior concerning their Young’s modulus, ultimate tensile strength and tensile elongation at fracture of the studied membranes was also investigated [29]. These three characteristic mechanical properties were evaluated from the tensile axial stress–strain curves, and their full calculations have been described in our previous work [43].

**Figure 7:**
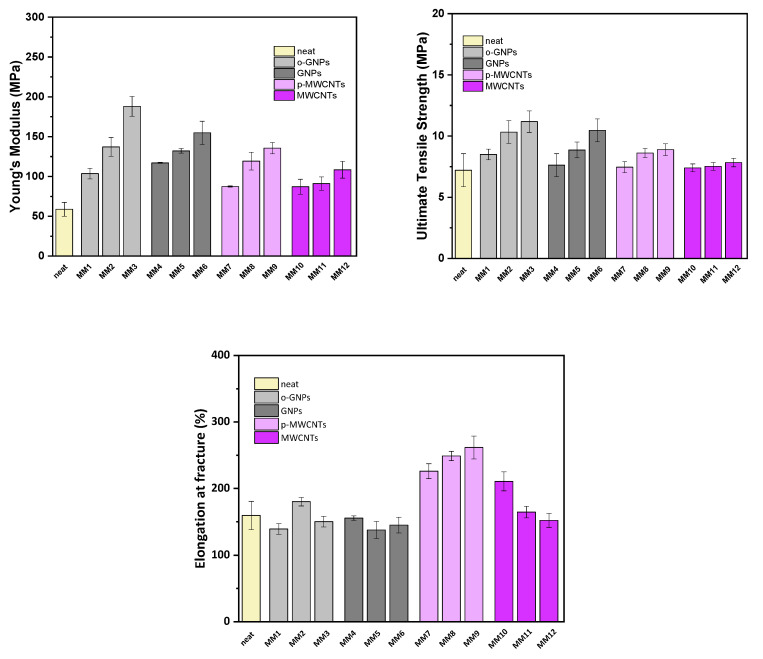
The mechanical behavior (tensile properties) concerning the Young’s modulus, ultimate tensile strength and tensile elongation at fracture, as calculated from corresponding stress–strain curves of all studied flat sheet Pebax-1657-based membranes.

In Table 3 and Figure 7, the numerical results of the mechanical tests of all samples are summarized, and the tensile properties of all membranes are depicted. The measurements were performed at ambient humidity of about 50%, whereas the samples were pre-equilibrated at this condition prior to each measurement. By analyzing the data in Table 3, it becomes clear that the values for the twelve MMMs can differ slightly or significantly compared to the “neat” membrane. The characteristic factor, which plays the major role in the determination of the Young’s modulus and ultimate tensile strength properties, is the concentration of the nanofiller material. In all four cases of different added nanofiller materials, higher concentration results in higher values of both abovementioned properties. For both properties, the addition of GNPs is more effective than the addition of MWCNTs. The highest values are reached in the cases of oxidized GNPs and plasma-treated MWCNTs and not for the corresponding raw materials. Specifically, for the membranes prepared with oxidized GNPs, the Young’s modulus increased up to 3.2 times (MM3) and for membranes with the plasma-treated MWCNTs up to 2.3 times (MM9) compared to the neat polymeric membrane. The observed differences between the mixed matrix membranes with raw and modified nanofillers are explained by the homogeneous and uniform dispersions of the modified GNPs and MWCNTs in the polymeric matrix, leading to stiffness enhancement of the polymer composite.

The Young’s modulus value, ~59 MPa, of the neat Pebax-1657 membrane is also reported by Duan et al. [44] in their recent work, where covalent organic frameworks (COFs)-functionalized MMMs were studied concerning CO_2_/N_2_ performance. Similar to our results is the behavior of the functionalized-GO/Pebax-1657 membranes in the work of Zhang et al. [45], where the addition of functionalized graphene oxide (f-GO) into the Pebax-1657 matrix resulted in higher Young’s modulus values, from ~46 MPa for the neat membrane up to 126 MPa for the 0.7 wt.% f-GO/Pebax-1657 sample. In contrast to our results, where the addition of carbon nanofillers led to the increase in the Young’s moduli, for the reported addition of COF-5 at concentrations up to 3 wt.%, the Young’s modulus was always subordinate to the corresponding neat membrane. This behavior of the Young’s modulus detriment has also been observed in another work of Fam et al. [46]. Indeed, for the case of Pebax-1657/ionic liquid (IL) membranes, the initial value of 73.7 MPa was decreased down to 1.2 MPa for the membrane with 80% of IL loading.

Furthermore, the good interfacial adhesion between the polymer and the nanofillers impelled the high toughness of the mixed matrix membranes, and again the ones with embedded treated nanofillers presented better results. Then, the ultimate tensile strengths of MM3 and MM9 membranes were higher than those of the neat membrane by 55.3% and 23.5%, respectively.

On the other hand, the more flexible MWCNT nanofillers enhanced the membranes’ elongation at fracture more effectively than the stiffer GNPs nanofillers. An increase up to 64.1% in the elongation at fracture was observed for the 5.3 wt.% plasma-treated MWCNTs fraction (MM9 membrane) compared to the neat polymeric membrane. In contrast, a maximum elongation (210.8%) was achieved for MM10 membrane (0.7 wt.% raw MWCNTs), and a further increase in raw MWCNTs content up to 5.3 wt.% impaired the elongation.

This trend could be explained based on the hypothesis that at high MWCNT loadings, the membranes (MM11 and MM12) become more brittle because of a weaker interfacial binding between polymer and nanofiller and simultaneously a higher restriction of the chains’ mobility within the polymer matrix [47,48]; conversely, the stronger interaction between the modified MWCNTs and the polymer matrix caused by the incorporation of functional groups in the nanofiller’s structure results in a better outcome.

The trade-off between ductility and tensile strength has also been reported in literature [49,50]. The rigid agglomerates of MWCNTs in the soft segment of the polymer matrix, forming after an inefficient dispersion, act as stress raisers resulting in premature fracture. 

An overall remark is that the type of nanofiller, its modification/treatment (which facilitates its good dispersibility), as well as its weight fraction in the polymer matrix significantly affect the mechanical properties of the prepared mixed matrix membranes.

In order to compare the experimental Young’s modulus and tensile strength of the mixed matrix membranes with theoretical predictions, three (Halpin–Tsai, Ekvall and Whitney–Riley) and two (Halpin–Kardos and Hirsch) well-known models were employed, respectively. Firstly, for the aligned and randomly and unidirectionally distributed filler conditions, the equations of the Halpin–Tsai model are the following [51]:

E_c_ = E_m_ [3/8 × (1 + n_L_ζφ_f_/1 − n_L_φ_f_) + 5/8 × (1 + 2n_T_φ_f_/1 − n_T_φ_f_)] (random) (2)

E_c_ = E_m_[(1 + n_L_ζφ_f_/1 − n_L_φ_f_)] (aligned) (3)

n_L_ = (E_f_/E_m_ − 1)/(E_f_/E_m_ + ζ) (4)

n_T_ = (E_f_/E_m_ − 1)/(E_f_/E_m_ + 2) (5)

ζ = k(l_f_/t_f_) (6)

where E_c_, E_m_ and E_f_ are the Young’s moduli of the composite, the Pebax matrix and the filler (MPa), respectively; φ_f_ is the volume fraction of the filler in the composite, derived from the equation φ_f_ = (w_f_/ρ_f_)/(w_f_/ρ_f_ + (1 − w_f_)/ρ_m_, where w_f_ is the filler mass fraction and ρ_f_ and ρ_m_ the densities of the filler (2.2 and 2.0 g/cm^3^ for GNPs and MWCNTs, respectively) and the matrix (1.01 g/cm^3^), respectively; ζ is a parameter regarding the nanofiller’s geometry, distribution and loading with k = 2/3 and 2 for GNPs and MWCNTs, respectively; while l_f_ and t_f_ refer to the length and thickness/diameter of GNPs/MWCNTs, respectively. These latter parameters were defined by SEM analysis.

According to the Ekvall model, the Young’s modulus is calculated using the following equations [52]:

E_c_ = E_f_E’_m_/[E’_m_φ_f_ + E_f_(1 − ν_m_^2^)φ_m_] (7)

E’_m_ = E_m_/(1 − ν_m_^2^) (8)

where φ_m_ is the volume fraction of the matrix in the composite and ν_m_ is the Poisson’s ratio of the matrix (0.3).

Moreover, for the Whitney–Riley model, the Young’s modulus can be calculated from the following equations [53]:

K_f_ = E_f_(1 − ν_f_ − 2ν_f_^2^) (9)

K_m_ = E_m_(1 − ν_m_ − 2ν_m_^2^) (10)

K_c_ = [(K_f_ + G_m_)K_m_ − (K_f_ − K_m_)G_m_φ_f_]/[(K_f_ + G_m_) − (K_f_ − K_m_)φ_f_] (11)

E_L_ = E_m_ + (E_f_ − E_m_)φ_f_ (12)

ν_yz_ = ν_f_φ_f_ + ν_m_(1 − φ_f_) (13)

L_m_ = 1 − ν_m_ − 2ν_m_^2^ (14)

L_f_ = 1 − ν_f_ − 2ν_f_^2^ (15)

ν_c_ = ν_m_ − 2(ν_m_ − ν_f_)(1 − ν_m_^2^)E_f_φ_f_/E_m_(1 − φ_f_)L_f_ + [L_m_φ_f_ + (1 + ν_m_)]E_f_ (16)

E_c_ = 2K_c_(1 − ν_yz_)E_L_/(E_L_ + 4K_c_ν_c_^2^) (17)

where ν_f_ is the Poisson’s ratio of the filler (0.22 for GNPs and 0.35 for MWCNTs), and G_m_ is the shear modulus of the matrix calculated from the equation G_m_ = E_m_/2(1 + ν_m_) (22.6 MPa). For all aforementioned models, the Young’s moduli of matrix (Pebax), GNPs and MWCNTs are 58.7 MPa (from the tensile test), ~1000 GPa and ~600 GPa, respectively.

**Figure 8:**
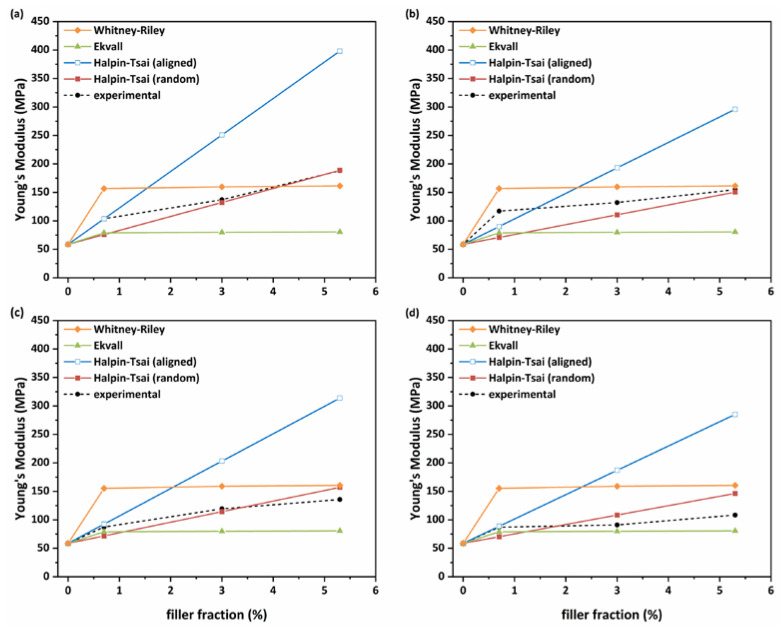
Comparison of the Young’s modulus as theoretically calculated from the Halpin–Tsai, Ekvall and Whitney–Riley models and from the experimental data for MMMs with (**a**) oxidized GNPs; (**b**) raw GNPs; (**c**) plasma-treated MWCNTs and (**d**) raw MWCNTs.

In Figure 8, the comparison of the theoretical calculations from the three models and the experimental data of the tensile moduli of all MMMs is depicted. For low nanofiller loading (0.7 wt.%) the Halpin–Tsai model for the aligned distributed nanofiller condition of all nanofillers, except raw GNPs, fitted experimental data perfectly. For higher loadings, the Halpin–Tsai model for randomly and unidirectionally distributed nanofillers is most suitable, mainly for the case of modified nanofillers, indicating the quality of a sufficient dispersion and the avoidance of aggregate formation. In general, the other two models, that are based only on Poisson’s ratio, diverge from experimental data to a greater or lesser extent, depending on the type or amount of nanofiller.

Similarly, for the theoretical calculations of the composites’ ultimate tensile strength, the Halpin–Kardos model is based on the following equations [54]:

n = (UTS_f_/UTS_m_) − 1/(UTS_f_/UTS_m_) + ζ (18)

UTS_c_ = UTS_m_(1 + ζnφ_f_/1 − nφ_f_) (19)

where UTS_f_, UTS_m_ and UTS_m_ are the ultimate tensile strengths of the filler, matrix and composite (MPa), respectively. Finally, the equation of the Hirsch model is given below [55]:

UTSc = x(UTSmφm + UTSfφf) + (1 − x)UTSfUTSm/(UTSmφf + UTSfφm)(20)

where x is a parameter that determines the stress transfer between the matrix and the filler. For both abovementioned models, the tensile strengths of the Pebax matrix, GNPs and MWCNTs are 7.2 MPa (from the tensile test), ~10 GPa and ~20 GPa, respectively. 

**Figure 9:**
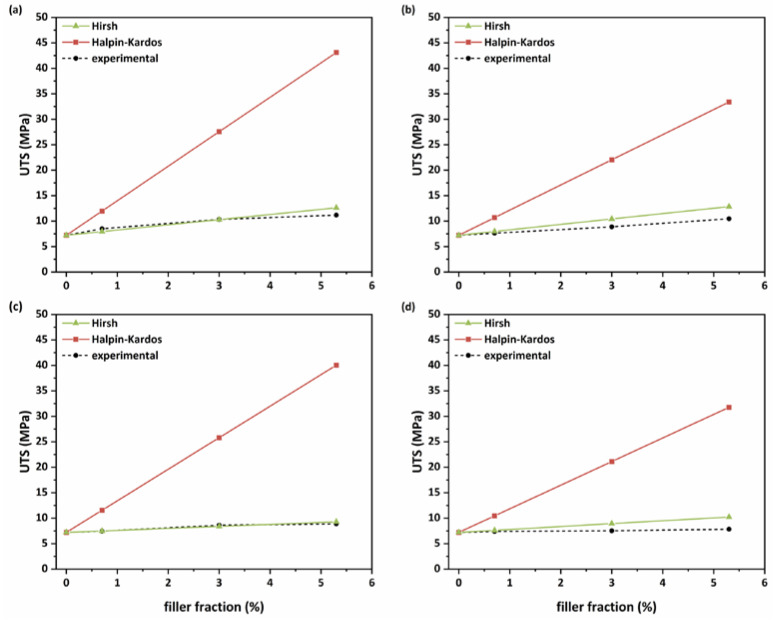
Comparison of the theoretical and experimental tensile strengths of the MMMs with (**a**) oxidized GNPs; (**b**) raw GNPs; (**c**) plasma-treated MWCNTs and (**d**) raw MWCNTs.

The overall theoretical calculations are illustrated in Figure 9. As observed, the Hirsch model is apparently more consistent for all types of nanofillers than the Halpin–Kardos model, indicating that parameter x (Equation (20)) is a crucial factor for the prediction of the tensile strength of the composites.

### 3.5. CO_2_/CH_4_ Permeability and Selectivity Results

In the present work, the binary CO_2_/CH_4_ mixture of gases was selected in order to study the membranes’ performance based on their permeability and selectivity. Experiments were conducted over a feed pressure range of 1.3 to 5.0 bar at the temperature of 25 °C. The measurements were performed as described in Section 2.4. In Table 4, the CO_2_ and CH_4_ permeability values and the respective CO_2_/CH_4_ selectivities for the 10/90 (molar concentrations) CO_2_/CH_4_ gas mixture are presented at five studied feed pressures of 1.3, 2, 3, 4 and 5 bar.

**Figure 10:**
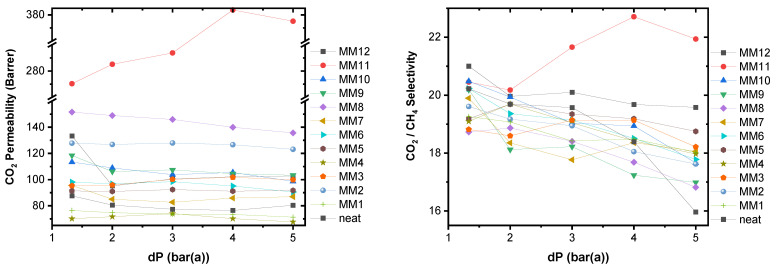
CO_2_ permeability (left) and CO_2_/CH_4_ separation factor (selectivity) (right) versus pressure drop across all studied MMMs for 10% *v*/*v* CO_2_ in CH_4_. Temperature was kept constant at 298 K.

Table 4 and Figure 10 exhibit the effect of both GNPs and MWCNTs as four different nanomaterial types for three different filler-loading concentrations. All membranes are selective for CO_2_ and present permeability values fluctuating between 67 (MM4 membrane at 5 bar feed pressure) and 384 Barrer (MM11 membrane at 4 bar feed pressure).

As seen in Table 4, the CO_2_ permeability values change in a different manner for the MWCNTs and the GNPs-based MMMs. For the cases of raw and oxidized GNPs (samples MM1 and MM4), a decrease of ~10% in CO_2_ permeability is observed for the MMMs loaded with 0.7 wt.% o-GNPs. This can be attributed to the fact that this very low number of GNPs works like a crosslinker material and makes the polymeric matrix less flexible with lower free volume than the neat matrix, resulting in a decrease in gas diffusivity and consequently also gas permeability [56]. By increasing the number of GNPs, the CO_2_ permeability also increases, presenting a maximum value for the concentration of 3 and of 5.3 wt.% in the cases of oxidized and raw GNPs, respectively. This behavior is in correlation with what is reported in literature for Pebax [46,57,58] but also for other polymeric mixed matrix membranes [16,59,60]. Here, it must be noted that the addition of the oxidized GNPs finally provides improved properties and better position in the Robeson plot of selectivity versus permeability, as the selectivity is remaining constant, and simultaneously the CO_2_ permeability increases about 46% at 1.3 bar.

On the other hand, in the case of MWCNTs-based MMMs the CO_2_ permeability increases even for the very low amount of 0.7 wt.%, whereas GNPs require concentrations above 3 wt.% in order to increase permeability, as mentioned. The smooth one-dimensional nanochannels of the MWCNTs could act as accelerated CO_2_ transport pathways through the MMMs. For both raw and plasma-treated carbon nanotubes, and at all feed pressures, higher CO_2_ permeability is observed in the case of MMMs with filler concentration of 3 wt.%. Furthermore, the membranes with oxidized GNPs need a concentration of 5.3 wt.% in order to reach MWCNT performance concerning permeability.

Effect of Feed Pressure: All membranes were tested in CO_2_/CH_4_ (10/90 vol.%) at five different transmembrane pressures, 1.3, 2, 3, 4 and 5 bar. With one exemption (that of the MM11 membrane), for the twelve membranes the influence of feed pressure on CO_2_ permeability was negligible. As seen in Table 4 and Figure 10, only small fluctuations, of about ±2–7%, in CO_2_ permeability were observed as pressure rose from 1.3 to 5 bar. However, for the MM11 membrane, the effect of feed pressure on CO_2_ permeability was positive, with an increase from 270 Barrer at feed pressure 1.3 bar to 384 Barrer at feed pressure of 4 bar.

On the contrary, the effect of pressure on CO_2_/CH_4_ selectivity is negative with a slight decrease compared to the value of the neat membrane. The only exception to this trend was observed for the M11 membrane (see Figure 10). At this point, irrespective of their concentration, nanofillers retain the selectivity of the neat membrane above 4 bar, withstanding membrane compaction. Overall, MMMs exhibited enhanced gas permeabilities with up to fivefold values without sacrificing selectivity compared to the neat polymeric membrane.

## 4. Conclusions and Outlook

Pebax-1657 mixed matrix flat sheet membranes were prepared following the solution casting/solvent evaporation technique, characterized and tested concerning their CO_2_/CH_4_ separation performance. Two types of carbon nanofillers were used, namely multi-walled carbon nanotubes and graphene nanoplatelets, both as raw materials but also after plasma treatment modification and oxidation process, respectively. In both cases, the modified nanomaterials (the oxidized GNPs and the plasma-treated MWCNTs) had a stronger reduction effect on water wettability of the prepared MMMs. The addition of these carbon-based nanomaterials resulted in membranes with improved mechanical properties and higher CO_2_ permeability, while selectivity was maintained at the level of the neat membrane. The polar groups of modified nanofillers form hydrogen bonds with the polymeric chains of Pebax, and hydrogen bonding interactions between nanofillers and Pebax are developed, resulting in the polymer chain packing disturbance and increase in free volume (voids) for the CO_2_ and CH_4_ molecules penetration and consequently the increase in gas diffusion (permeability). Furthermore, the functional groups on the surface of nanofillers may interact with gases (e.g., CO_2_) and increase the solubility in the MMMs, as the CO_2_ gas transition through the MMMs is facilitated. Improved withstanding of membrane compaction was an additional benefit of all examined nanofillers. The CO_2_/CH_4_ separation factor of the tested mixture of 10/90 (mole fraction) fluctuated between 16 and 21, with the higher values being observed at the lowest transmembrane pressure of 1.3 bar. A general conclusion is that MMMs exhibited enhanced gas permeabilities up to fivefold values (~384 Barrer for MM11) without sacrificing the gas selectivity in comparison with the neat membrane.

## Figures and Tables

**Table 1:** Concentrations of precursor solutions, polymer and carbon nanomaterials (CNMs) and final membrane concentrations after drying for the three studied cases of filler content.

Case	Polymer in Solution A	Filler in Solution B	Concentration in Final Solutions (A + B)		Filler Concentrations in MMMs
			Polymer	Filler	
“I”	5	0.052	3.0	0.021	0.7
“II”	5	0.225	3.0	0.090	3.0
“III”	5	0.398	3.0	0.159	5.3

All percentages in wt.%.

**Table 2:** Water contact angle measurements of the studied flat sheet Pebax-1657-based membranes.

Sample	Neat	MM1	MM2	MM3	MM4	MM5	MM6	MM7	MM8	MM9	MM10	MM11	MM12
Average WCA (^ο^)	**63.4**	83.0	85.0	**107.8**	76.0	78.0	**86.0**	69.0	76.6	**87.0**	68.9	70.5	**71.0**

with bold are mentioned the higher WCA values of each membrane group.

**Table 3:** Mechanical properties of the studied flat sheet Pebax-1657-based membranes.

Sample	Filler Material	Filler Fraction (%)	Young’s Modulus (MPa)	Ultimate Tensile Strength (MPa)	Elongation at Fracture (%)
**neat**	**---**	---	58.7 ± 8.8	7.20 ± 1.35	159.5 ± 21.3
**MM1**	**Oxid. GNPs**	0.7	103.6 ± 6.6	8.50 ± 0.44	139.2 ± 8.0
**MM2**		3.0	137.2 ± 11.9	10.33 ± 0.93	**180.2 ± 6.4**
**MM3**		5.3	**188.0 ± 8.8**	**11.18 ± 0.87**	150.1 ± 8.0
**MM4**	**Raw GNPs**	0.7	117.0 ± 0.7	7.62 ± 0.94	**155.5 ± 3.3**
**MM5**		3.0	132.0 ± 2.9	8.86 ± 0.64	137.7 ± 12.9
**MM6**		5.3	**154.8 ± 14.6**	**10.47 ± 0.93**	145.0 ± 11.9
**MM7**	**Plasma MWCNTs**	0.7	87.3 ± 0.8	7.46 ± 0.45	226.0 ± 11.2
**MM8**		3.0	119.3 ± 11.1	8.61 ± 0.37	249.0 ± 7.0
**MM9**		5.3	**135.6 ± 7.1**	**8.89 ± 0.48**	**261.7 ± 17.2**
**MM10**	**Raw MWCNTs**	0.7	87.0 ± 9.5	7.40 ± 0.32	**210.8 ± 14.2**
**MM11**		3.0	91.0 ± 8.2	7.53 ± 0.32	164.5 ± 8.7
**MM12**		5.3	**108.4 ± 10.6**	**7.84 ± 0.35**	152.0 ± 10.4

Underline values are referred to the reference material, the neat membrane. Bold values are referred to the higher values of each membrane group.

**Table 4:** CO_2_ permeability (Barrer) and CO_2_/CH_4_ selectivity values for the thirteen studied membranes for 10% *v*/*v* CO_2_ in CH_4_.

Sample	Filler Material	% Concentr.	CO_2_ Permeability (Barrer) at					CO_2_/CH_4_ Selectivity at				
			1.3	2	3	4	5 (bar)	1.3	2	3	4	5 (bar)
**neat**	**---**	---	87.6	80.5	77.4	76.5	80.4	20.2	19.7	19.6	18.4	16.0
**MM1**	**Oxid. GNPs**	0.7	76.5	74.8	73.6	73.4	71.4	19.3	19.0	18.4	18.5	18.0
**MM2**		3.0	127.8	126.8	128.0	126.6	123.1	19.6	19.2	18.9	18.0	17.6
**MM3**		5.3	95.3	95.6	100.2	101.8	99.9	18.8	18.6	19.1	19.1	18.2
**MM4**	**Raw GNPs**	0.7	70.1	71.7	74.0	70.2	67.7	19.0	19.7	19.0	18.4	18.0
**MM5**		3.0	91.4	91.0	92.4	91.2	91.8	19.2	19.7	19.3	19.2	18.7
**MM6**		5.3	98.1	96.7	98.4	95.1	90.6	20.2	19.4	19.1	18.5	17.8
**MM7**	**Plasma MWCNTs**	0.7	95.6	85.0	82.7	85.9	86.9	19.9	18.4	17.8	18.4	18.0
**MM8**		3.0	151.6	148.9	145.9	140.0	135.6	18.7	18.9	18.4	17.7	16.8
**MM9**		5.3	118.4	106.2	107.4	104.5	103.3	20.2	18.1	18.2	17.2	17.0
**MM10**	**Raw MWCNTs**	0.7	113.4	108.9	103.8	105.4	98.7	20.5	19.9	19.0	18.9	17.7
**MM11**		3.0	270.3	285.1	293.8	383.7	375.1	20.5	20.2	21.7	22.7	21.9
**MM12**		5.3	133.3	95.2	100.5	102.2	102.7	21.0	20.0	20.1	19.7	19.6

## Data Availability

Not applicable.
